# Personalized Management of Patients with Proliferative Diabetic Vitreoretinopathy

**DOI:** 10.3390/life14080993

**Published:** 2024-08-09

**Authors:** Monika Ecsedy, Dorottya Szabo, Zsuzsa Szilagyi, Zoltan Zsolt Nagy, Zsuzsanna Recsan

**Affiliations:** Department of Ophthalmology, Semmelweis University, 1085 Budapest, Hungary

**Keywords:** proliferative diabetic retinopathy, pars plana vitrectomy, HbA1c, visual acuity, tractional macular detachment

## Abstract

**Purpose:** To evaluate prognostic factors for visual outcome in patients with diabetes who have undergone vitrectomy (PPV) for severe proliferative diabetic vitreoretinopathy (PDVR) in at least one eye in the past 15 years. **Methods:** Medical records of 132 eyes of 66 patients were analyzed (median age 52 years 21–80; patients with type 1/2 diabetes 40/26; median follow-up 38 months 9–125). Correlations between final favorable visual outcome defined as 0.5≤ best-corrected visual acuity (BCVA) and prognostic factors (age, sex, type and duration of diabetes, metabolic status, BCVA, diabetic retinopathy status, data of preoperative management, data of vitrectomy, and postoperative complications) were analyzed. **Results:** BCVA improved significantly in the entire study cohort (from median 0.05 min–max 0.001–1 to 0.32, 0.001–1, *p* < 0.001). Visual stabilization was achieved in the majority of patients, and good visual acuity (0.5 ≤ BCVA) was maintained in more than one-third of the eyes. Multivariable GEE statistics showed that in addition to the duration of diabetes and stable HbA1c values, only preoperative tractional macular detachment proved to be an independent significant predictor of visual outcome. **Conclusions:** Pars plana vitrectomy is a useful tool when performed early before tractional macular detachment. However, long-term visual stability can only be achieved with good metabolic control.

## 1. Introduction

Diabetic retinopathy (DR) continues to be the leading cause of blindness among working-age adults in developed countries, despite advances in screening techniques and treatment options. It is estimated to affect around 90 million of the 463 million people living with diabetes mellitus worldwide [1]. The most important sight-threatening complication is proliferative retinopathy (PDR), which will develop in approximately 50% of type 1 and 25% of type 2 patients after 20 years of duration, according to the Wisconsin Epidemiological Study [2]. Obviously, the primary goal is to prevent complications. Recent advances in diagnostic modalities such as wide-field OCT, or hyperspectral imaging, enable easier and earlier detection of patients with high-risk retinopathy [3,4].

Effective treatment options include panretinal laser photocoagulation (PRP), intravitreal anti-vascular endothelial growth factor (anti-VEGF) therapy, and pars plana vitrectomy (PPV).

While PRP has been the traditional method for the prevention of severe vision loss for many years, and anti-VEGF injections have been shown to provide an effective option for patients with PDR, it is important to note that both therapies carry a significant treatment burden and may require multiple retreatments. According to the Early Treatment of Diabetic Retinopathy Study (ETDRS), PRP can reduce the occurrence of severe visual impairment by 50%. However, in 15% of PDR cases, vitrectomy may be required due to non-resolving vitreous hemorrhage, tractional retinal detachment, or combined tractional and rhegmatogenous retinal detachment [5,6]. It is also important to note that 33% of -eyes treated with anti-VEGF and then lost to follow-up may develop tractional retinal detachment [7,8].

Since Robert Machemer performed the first PPV in a patient with PDR in 1973 to treat non-clearing vitreous hemorrhage, vitrectomy has undergone significant development and modernization, leading to a broadening of the indications for vitrectomy and improved outcomes. Vitrectomy in patients with diabetes is usually performed in cases of tractional retinal detachment (TRD) involving the posterior pole and non-clearing vitreous hemorrhage. Benefits were also seen for progressive fibrovascular proliferation, ghost cell glaucoma, and traction-induced diabetic macular edema (DME) [2,5]. The outcomes of vitreous surgery have improved significantly due to several advancements in the field. These include the introduction of wide-angle visualization, small-diameter instruments with high cutting speeds and precise pressure control, the use of anti-VEGF drugs, and the ability to perform endolaser coagulation. The benefits of early vitrectomy, especially in younger patients with an attached posterior hyaloid, include the preservation of vision, reduced treatment burden, and fewer subsequent treatments [7,9]. However, the role of pars plana vitrectomy PPV in the treatment algorithm for proliferative diabetic retinopathy (PDR) remains controversial. A better understanding of the real-world long-term outcomes of vitrectomy for PDR could enhance clinical management and visual outcomes. Advanced BCVA outcomes may lead to an improved quality of life for patients with advanced diabetic complications who may be at risk of losing their independent ability to work and live.

The aim of the study was to analyze factors that may influence the maintenance of favorable visual acuity. Patients in this study underwent vitrectomy for severe proliferative diabetic retinopathy in one eye within 2–4 weeks of their first visit to our tertiary care center. The fellow eye underwent vitrectomy or was spared surgery with a personalized treatment plan including panretinal laser coagulation or intravitreal anti-VEGF injections, depending on its condition (i.e., preproliferative status, proliferative diabetic retinopathy, or proliferative diabetic vitreoretinopathy).

## 2. Materials and Methods

### 2.1. Standard Protocols, Approval, and Data Availability

Patients included in our retrospective study underwent pars plana vitrectomy for complications related to PDR at least once in one eye while being examined and treated at the Department of Ophthalmology, Semmelweis University, Budapest, Hungary between 2008 and 2022. The data were extracted from the Medsol system. The study was approved by the Institutional Ethics Review Board of Semmelweis University (SE RKEB 59/2023) and was conducted in accordance with the tenets of the Helsinki Declaration. The datasets are deposited in the Dryad database (doi:10.5061/dryad.4b8gthtnj).

### 2.2. Patients’ Clinical Data Analysis

Inclusion criteria were the following: (1) type 1 or type 2 diabetic patients who had undergone vitrectomy in at least one eye for severe PDR, (2) at least a 6-month follow-up period after the last surgery, and (3) information on metabolic control (at least the data at the first and last visit), ophthalmological status, details of the surgery, and other ophthalmological interventions in both eyes throughout the follow-up.

The main outcome measure was the final best-corrected visual acuity at the end of the follow-up.

The following data were collected: Baseline systemic parameters, which included the following: age, sex, type and duration of DM, hemoglobin A1c (HbA1c) level, known hypertension, and renal and cardiovascular complications. Regarding glycemic control, HbA1c levels were collected from the time of the first vitrectomy, and from the time of the last visits. In addition, the mean HbA1c was also calculated from the data available during the entire follow-up period. Patients with a GFR of less than 30 mL/min/1.73 m^2^ or who were under nephrological care or receiving hemodialysis were counted as patients with renal insufficiency. We also collected information on other serious systemic diseases such as uncontrolled hypertension, stroke, and cardiovascular (CV) complications, the latter including a history of acute ischemic heart disease or heart failure.

Baseline, intra- and postoperative ocular parameters included the first BCVA (when the patient first attended our department), preoperative and final BCVA (measured on the ETDRS chart), the status of DR (posterior hyaloid detachment, PVD; vitreous hemorrhage; tractional retinal detachment, TRD; and macular tractional detachment, MRD rubeosis), pre-/postoperative panretinal photocoagulation (PRP), pre- and postoperative anti-VEGF injections, epiretinal membranes, surgical techniques, intra- and postoperative complications, and the need for postoperative antiglaucoma treatment or cataract surgery. Depending on media opacity, ultrasonography and optical coherence tomography were used to assess posterior segment status. The morphology of the macula was assessed using the radial line algorithm of spectral domain optical coherence tomography (OCT, RTVue-XR Avanti, Optovue, Fremont, CA, USA). Fluorescein angiography was performed to detect any peripheral ischemia and proliferative foci.

### 2.3. Management

All first eyes and some of the fellow eyes underwent vitrectomy. Indications for vitrectomy were recurrent or non-resolving vitreous hemorrhage and/or tractional retinal detachment, tractional macular edema, and detachment. When a patient was first seen with severe bilateral PDR requiring surgery, the eye with the better visual prognosis was selected for vitrectomy. (The patients refused to undergo simultaneous single-session bilateral vitrectomy.) The 23-gauge 3-port pars plana vitrectomy was performed under regional or general anesthesia by two experienced vitreoretinal surgeons (RZ, EM). In cases of progressive lens opacities or anterior proliferative vitreoretinopathy, surgery was combined with phacoemulsification and posterior lens implantation. Triamcinolone acetate (TCA) was injected to stain the vitreous and facilitate the removal of the posterior hyaloid. Membranectomy or membrane peeling was performed bimanually under a chandelier light source.

Panretinal laser coagulation was added. Silicon oil (1300cst, SiO) or octafluoropropane (C3F8) was implanted as an endotamponade. In our cohort, preoperative anti-VEGF injections were used to treat central macular edema or as adjuvant therapy 3–5 days before PPV in cases of florid proliferation to prevent intraoperative bleeding. After surgery, uncomplicated cases received a combination of mydriatic, steroid, and non-steroid anti-inflammatory eye drops 3 times daily for 2 weeks.

The fellow eye was treated according to the stage of DR and individualized treatment was applied. Central macular edema was treated with anti-VEGF intravitreal injection five times in a monthly period, followed by pro re nata protocol. Intravitreal steroid implantation was also considered, especially in cases of persistent intraretinal fluid. In these cases, panretinal laser treatment was also recommended to the patients if extensive peripheral ischemia was detected on fluorescein angiography. In cases of severe nonproliferative or mild proliferative retinopathy, panretinal laser coagulation according to ETDRS protocol was the first choice of treatment. Anti-VEGF intravitreal injection was also administered as adjuvant treatment in cases of florid retinopathy or central macular edema.

The normalization of the patient’s metabolic parameters and the ophthalmic treatment occurred in parallel. Surgeons did not wait for CH metabolism to normalize as it was observed that this can take several months while retinopathy progresses rapidly. At the same time, it was considered important to maintain blood pressure at target levels before vitrectomy.

### 2.4. Statistical Analysis

Statistical analysis was performed using IBM SPSS Statistics 25 (SPSS Inc., Chicago, IL, USA). The Shapiro–Wilk’s test confirmed the non-normal distribution of data. Therefore non-parametric tests were applied. The main outcome measure was final visual acuity, and subanalysis was performed specifically for the group of eyes with favorable BCVA (0.5 ≤ BCVA) The eyes of the patients were divided into three groups, (a) primary vitrectomized eyes, (b) fellow eyes that also underwent vitrectomy during follow-up, and (c) non-vitrectomized fellow eyes.

Fisher’s exact test was used to compare categorical parameters. Comparisons were made using the Mann–Whitney U test for unpaired parameters and the Wilcoxon signed-rank test for paired pre- and postoperative parameters.

To detect any correlation between the parameters and the final BCVA, the non-parametric Spearman’s bivariate test was utilized. The effect of predisposing factors on the final BCVA was assessed through multivariable analysis using the general estimating equation (GEE) model. This test takes into account the within-subject correlation of parameters (right vs. left eye) by considering inter-eye correlations and between-visit correlations of parameters as repeated measurements.

In addition, the inclusion of both ocular and non-ocular risk factors as covariates, in general, by estimating equation models, allows for simultaneous control for their effect on the dependent variables. Covariates assessed as potential confounding factors, based on a priori hypotheses, included age, duration of diabetes, HbA1c level, hypertension, and retinopathy status (tractional macular detachment, tractional retinal detachment elsewhere, preoperative glaucoma, rubeosis, and best-corrected visual acuity at first visit).

## 3. Results

### 3.1. Descriptive Statistics and Comparisons

#### 3.1.1. Patients’ Characteristics

Medical records of 73 consecutive patients with PDR were analyzed, and seven patients were excluded due to a lack of regular HbA1c data.

All of the patients were referred to us due to severe proliferative retinopathy requiring vitrectomy in at least one eye. The data analyzed included 66 first vitrectomized eyes, 31 fellow eyes that also underwent vitrectomy during follow-up, and 35 non-vitrectomized fellow eyes. Eighteen patients (18/66, 27%) were younger than 40 years. The disease duration was at least 25 years in 25 patients (25/66, 38%). One patient was first diagnosed with diabetes during an evaluation for severe visual impairment.

**Figure 1:**
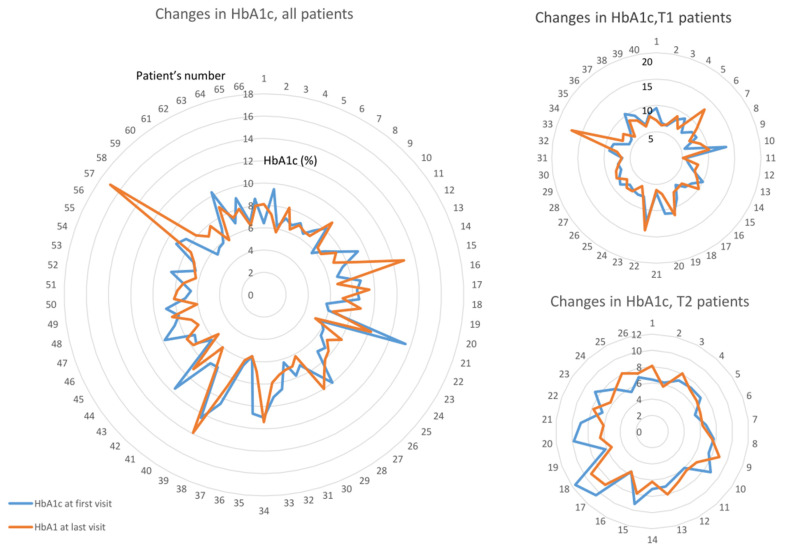
Comparison graphs of HbA1c data at first and last visits, respectively. Wilcoxon signed-rank test showed no significant differences between first and last HbA1c values, either for all patients or type 1 or type 2 groups.

At the first visit, patients with type 1 diabetes were found to be significantly younger, had a longer duration of diabetes, and had higher HbA1c levels compared to patients with type 2 diabetes. There were no discernible differences in other systemic parameters, such as sex, renal insufficiency, severe cardiovascular complications, or unstable blood pressure, between the two types of diabetes. Furthermore, we did not observe any significant differences in preoperative ophthalmological status between these groups. The baseline characteristics of our patients are summarized in Table 1, Figure 1 shows the changes in HbA1c. No significant changes were detected between the first and last HbA1c levels (Wilcoxon signed-rank test). Sometimes, the patients cancelled the appointment for a check-up or only came when they had a complaint, and the severe carbohydrate metabolism was then revealed. At the initial clinical eye examination, 44 patients (44/66; 66%) had uncontrolled hypertension, cardiovascular complications, or severe renal dysfunction (Table 1).

#### 3.1.2. Surgery

A total of 157 vitrectomies were performed in our study population, in 97 eyes of 66 patients (97/132 eyes, 73%) (median value, min–max 1; 1–4). Of the whole cohort, 54 eyes (55.67%) underwent only one vitrectomy, while 43 eyes (44.33%) required multiple vitrectomies. Silicone oil was applied in 58 eyes, and silicone oil explantation was also counted as an additional surgery. Combined phacoemulsification–vitrectomy was performed in 30 eyes (30/97 vitrectomized eyes, 31%). A posterior hyaloid detachment was confirmed during the surgery in 29 eyes (29/97 vitrectomized eyes, 30%), mainly in type 1 patients with diabetes (20 versus 9 eyes in type 1 or type 2 diabetics, respectively; Fisher’s test, *p* value = 0.1). The first eye underwent a vitrectomy within a month after first attendance. A total of 31 patients (47%, 31/66 patients, type 1 diabetes: 17; type 2 diabetes: 14) required surgery on both eyes. The median interval between the first and second eye surgeries was 2 months (min–max: 1–33). A total of 15 eyes (48%, 15/31 patients) underwent surgery within 3 months.

Severe intraoperative complications occurred in four eyes with tractional retinal detachment. During membrane peeling, retinal tears developed in three legally blind eyes, resulting in rhegmato-tractional retinal detachment and blindness. The fourth patient was on monoclonal antibody anticoagulation for atrial fibrillation, which was discontinued 24 h before surgery. During the otherwise uneventful surgery, an extensive subchorioidal hemorrhage involving the posterior pole developed resulting in legal blindness.

Postoperative complications: In the early postoperative period, bleeding into the vitreous cavity was the most common complication (38 eyes; 38 eyes/157 vitrectomies, 24%), which resolved in all cases within 1–3 months. At the end of the follow-up, combined antiglaucoma eye drop therapy was required for 39 eyes (39/132, 29%). Out of these patients, 10 eyes developed refractory glaucoma, 2 eyes underwent shunt implantation, and the others underwent cyclophotocoagulation.

At the end of the follow-up, active neovascularization was found on the iris in 10 eyes and on the disc or elsewhere on the retina in 5 eyes (15/132 eyes 11%). Tractional retinal detachment was described in nine eyes, and among them, all but one eye became blind or legally blind. Foveal center-involved persistent intraretinal fluid was observed in 12 eyes despite treatment with anti-VEGF intravitreal injection and intravitreal steroid implant. At the end of the follow-up, persistent intraretinal fluid was observed in four eyes, and another two eyes showed lamellar hole formation on the OCT scan. Apart from the cases mentioned above, no rhegmatogeneous retinal detachment has developed in any of the other eyes.

Changes in ophthalmological parameters in the three study groups are summarized in Table 2.

#### 3.1.3. Functional Results

**Figure 2:**
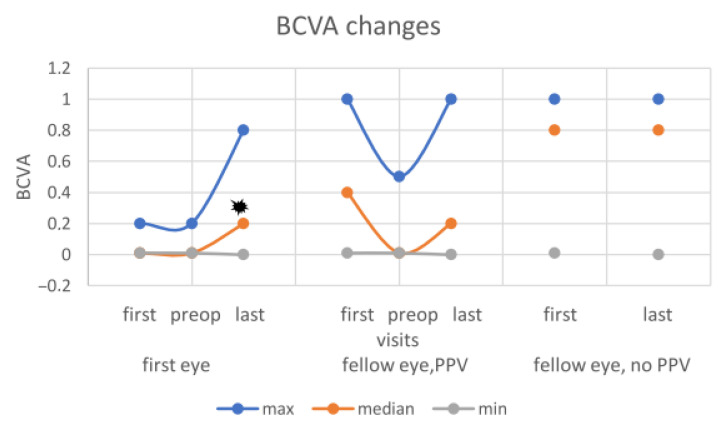
Changes in the median (min–max) best-corrected visual acuity (BCVA) in the three subgroups (preop: preoperative visit, on the day of surgery). The star indicates significant changes between BCVA at the first and final visits, respectively. The BCVA at first referral was preserved in fellow eyes.

Best-corrected visual acuity improved significantly in the entire study cohort (from median 0.05 min–max 0.001–1 to 0.32 0.001–1, *p* < 0.001). However, when analyzing the three study groups, only the first vitrectomized group showed a significant improvement (from median 0.04 min–max 0.01–1 to 0.2 0.001–1, *p* < 0.001). In the group of vitrectomized fellow eyes and non-vitrectomized fellow eyes, the observed changes were not significant (Figure 1). Favorable final BCVA was maintained in 53 eyes (53/132, 40%). At first attendance, 88 eyes (66%, 88/132) were legally blind, and BCVA improved to equal or better than 0.5 in 22 eyes (22/88, 25%). In total, 52 eyes (52/88, 59%) remained legally blind. Changes in BCVA in the three study groups are summarized in Figure 2.

Of the 97 vitrectomized eyes, 83 eyes (85%, 85/97) were legally blind at the first visit. A good final BCVA was achieved in 30 eyes (30%, 30/97; out of them, 21 were legally blind). At the final visit, 43% (43/97) of the eyes were legally blind.

In the group of first vitrectomized eyes (66 eyes of 66 patients), the majority were legally blind at baseline (64 eyes, 64/66, 96%). Of those, 18 eyes (28%,18/64) had a BCVA ≥ 0.5, and 26 eyes (26/64, 40%) remained legally blind after surgery.

In the group of fellow eyes that underwent vitrectomy (31 eyes), out of the 19 legally blind eyes (61%, 19/31), only 3 eyes had a final good BCVA. Good BCVA was maintained in 11 eyes (35%, 11/31).

Out of the thirty-five non-vitrectomized fellow eyes, three were kept for observation as we believed that intervention would not be beneficial. Two of these eyes had extensive tractional retinal detachment and were nearly blind, while the third had a burned-out TRD nasal to the disc and a BCVA of 1.0. The remaining thirty-two eyes were treated with a panretinal laser, anti-VEGF injections, or steroid implants.

### 3.2. Univariate Analysis

#### 3.2.1. Potential Factors Affecting Final BCVA

The results of the univariate statistical analysis indicate that several general and ophthalmological factors may have a significant impact on good visual acuity at the end of the follow-up. These factors are summarized in Table 3. Notably age, disease duration, and average HbA1c level showed a strong correlation with favorable BCVA outcomes while follow-up had no impact.

#### 3.2.2. Intercorrelations among Other Factors

A higher mean HbA1c level was linked to an increased risk of developing rubeosis (*p* = 0.014). Older age or type 1 patients had longer disease durations (for age, *p* = 0.037, correlation coefficient 0.181; for type 1, *p* < 0.001, correlation coefficient −0.360). Older age correlated with type 2 diabetes (*p* < 0.001, correlation coefficient 0.443). Younger age was associated with a higher mean HbA1c (*p* < 0.001, correlation coefficient −0.333) and more frequent renal insufficiency (*p* = 0.020, correlation coefficient −0.203). The frequency of tractional detachment was higher in younger patients (*p* < 0.001, correlation coefficient −0.357). The follow-up period showed no correlation with any parameter.

### 3.3. Multivariable Analysis for Predictors of Favorable Functional Outcome

In eyes with a favorable final visual acuity (BCVA ≥ 0.5), several factors were identified as potentially significant predictors. Multivariable analysis was performed to determine which factors had the most significant and independent effect on visual outcome, as multiple interactions between these factors were expected. According to multivariate analysis using the GEE method (quasi goodness of fit, 39,974), these factors are disease duration (*p* = 0.022), mean HbA1c (*p* = 0.002), and preoperative macular tractional detachment (*p* = 0.001).

## 4. Discussion

In the current study, which examined the long-term outcomes of patients who underwent vitrectomy for PDR or received personalized management over the past 15 years, our main findings were as follows: (a) PPV was found to be a viable option for improving visual function in patients with advanced PDR; (b) panretinal laser coagulation with the addition of intravitreal anti-VEGF injection is a well-established treatment for severe non-proliferative retinopathy and proliferative retinopathy without tractional detachment; (c) among the ophthalmological data, preoperative macular tractional detachment was the main predictor of the final visual outcome; and (d) of the general conditions, the disease duration and the average HbA1c level had the most significant influence on the final visual acuity. The majority of our patients achieved visual stabilization, with over half of them having favorable visual acuity (0.5 ≤ BCVA) in one eye. However, it is worth noting that two-thirds of the eyes that were legally blind at the beginning remained legally blind.

These findings are consistent with those reported in previous studies based on real-world experience. Early PPV may prevent the development of tractional retinal detachment, stabilize the fundus long-term, and reduce the number of complications and medical costs. Nowadays, early surgery is preferred because better postoperative visual acuity can be achieved when a vitrectomy is performed in the pregliotic phase [10]. Berrocal et al. demonstrated the beneficial effects of early PPV both in terms of postoperative BCVA and the rate of subsequent treatments required, when compared to patients who received PRP or PPV, over an 8-year follow-up period. The only disadvantage was the more rapid progression of cataracts in the PPV group [11]. The DRCR.net protocol AB compared aflibercept and early vitrectomy with PRP in the treatment of PDR with vitreous hemorrhage (VH). While no significant difference was reported in long-term BCVA outcomes over 24 weeks, early vitrectomy resulted in faster visual recovery and less recurrent vitreous hemorrhage [9,12].

Recently, the posterior hyaloid position has been suggested to play a crucial role in determining the appropriate approach to vitrectomy. Previous studies have also highlighted the impact of the posterior hyaloid on the progression of PDR earlier [13,14]. Younger patients, with attached or partially detached posterior hyaloid are more likely to progress to vitreous hemorrhage or TRD. Complete PVD in older patients may be a protective factor against the progression to PDR and TRD, as neovascular vessels can only grow on the surface of the retina in the absence of a vitreoretinal interface. Berrocal found in a retrospective real-world study that eyes with complete PVD can be treated with less aggressive PRP and anti-VEGF, but the more beneficial treatment for eyes with no or partial PVD may be PPV with hyaloid removal and less confluent PRP [11]. However, the detection of posterior hyaloid attachment can often be a challenge in patients with diabetes due to the frequent occurrence of vitreous schisis. In such cases, the posterior hyaloid is divided into multiple layers, and in our experience, its exact position can only be determined during surgery by injecting dye to enhance visualization of the vitreous dye. At the time of surgery, attached hyaloids were found in the majority of our patients, and as one of its beneficial aspects, vitrectomy has been shown to eliminate the traction caused by posterior hyaloids, which may prevent or stop the formation of tractional retinal detachment. Our study cohort showed that tractional retinal detachment reaching the macula was a major negative predictive factor for the final visual outcome. According to a recent meta-analysis by McCollough et al., patients with PDR and TRD have a high anatomic success rate of up to 90%, but despite this success, final postoperative visual acuity (VA) remains low, and the only significant factor for favorable visual outcome was good preoperative VA [15].

Adjuvant preoperative anti-VEGF injections can further improve surgical safety and reduce the incidence of intra- and postoperative complications, intraoperative bleeding occurs less frequently, the need for intraoperative endodiathermy decreases, and there are shorter surgery times, fewer iatrogenic retinal tears, and fewer early postoperative recurrent VH, or its faster absorption can be achieved [16,17]. With the preoperative administration of intravitreal anti-VEGF injections, the surgical technique has also become simpler, e.g., the membranes become easier to peel off. Zhao et al. also reported better postoperative BCVA after PPVs supplemented with adjuvant anti-VEGF treatment [18]. In our patient cohort, this method was frequently used and found to be effective in preventing severe intraoperative bleeding and facilitating membrane peeling. However, in cases of preoperative adjuvant treatment, it is important to remember to perform panretinal laser treatment during and after surgery, since the anti-VEGF administered during pretreatment only provides adequate VEGF inhibition for four weeks and most of it is removed during vitrectomy. As observed in our current study, the utilization of this technique did not have a significant effect on the final BCVA, nor did it reduce the risk of postoperative hemorrhage.

The most common postoperative complications in our study were rubeosis with secondary glaucoma, and vitreous hemorrhage (VH), which are in line with the literature. In the most severe cases, retinopathy progressed to anterior segment neovascularization which led to refractory stage secondary glaucoma, and Ahmed shunt implantation was the only effective long-term solution. Some patients experienced a severe increase in IOP during the early postoperative period due to posterior synechiae formation and pupillary block. In these cases, Nd-YAG iridotomy was not always successful because of bleeding from the rubeotic iris tissue.

The development of VH, observed in 24% of our cohort, is not uncommon during the postoperative weeks and months, as other studies have reported an incidence of 4–45% in PDR patients [19,20]. Motoda et al. reported that the most important predictor of bleeding in the late postoperative period is the duration of the surgery [21]. In addition, fibrovascular proliferation in the sclerotomy wound is also mentioned as one of the possible causes of bleeding [22]. The use of gas tamponades, cryotherapy of the sclerotomy sites, preoperative anti-VEGF treatment, and aggressive preoperative PRP treatment can significantly reduce the likelihood of postoperative VH [23]. In our experience, postoperative vitreous hemorrhage did not have a significant negative impact on final visual acuity as it usually resolved spontaneously or after anti-VEGF injection within three months after surgery. In cases where the hemorrhage persisted, we decided to perform another surgery to complete the panretinal laser coagulation and to prevent the formation of secondary rubeosis or ghost cell glaucoma.

Silicone oil is implanted in the most severe cases. In the long term, however, it has been linked to an increased risk of secondary glaucoma and neovascularization [23]. In our study cohort, we used SiO as an endotamponade when traction could not be completely removed, severe diffuse bleeding was observed during the surgery, or multiple retinal brakes were found in several locations. The use of SiO was not an independent risk factor for surgical failure in our patient cohort, but if we did not remove it within six months, or even by the end of the follow-up, valuable visual acuity could not be achieved.

In terms of general health, mean HbA1C levels and their values at the end of the follow-up were the only significant predictors of long-term valuable visual acuity preservation.

In our experience, PPV can assist our patients in gaining time to control their diabetes. Early PPV offers the opportunity to complete panretinal laser coagulation and prevent the development of macular tractional detachment. By eliminating tractional effects, a safer option of intravitreal anti-VEGF treatment is provided in these severe cases. We need to use all available therapeutic tools to try and keep the retinopathy “asleep” because until CH metabolism is normalized, retinopathy can progress, and vision is compromised. Although earlier surgery may be advantageous, the focus should be on identifying patients at a high risk of developing MRD and preventing it.

Data from the randomized multicenter studies as well as findings of recent publications on real-life experience with PPV in PDR are summarized in Supplementary Table S1.

Strengths of this study include the long duration of the follow-up, the relatively large sample size, and the collection of initial vitrectomy data at a single tertiary center, which reduces external sources of variability. In addition, we evaluated several possible confounding factors, such as metabolic status data, in addition to ophthalmological findings, providing a comprehensive assessment of the long-term outcomes of vitrectomy for PDR in real-world practice.

Limitations: A major limitation is the retrospective nature of the study, which did not allow for the standardization of measurements. Additionally, the heterogeneity of the study cohort and the multiple potential interactions between systemic and ophthalmological factors may complicate the interpretation of the results.

## 5. Conclusions

In conclusion, this study retrospectively analyzed patients who underwent vitrectomy for advanced PDR. Among the systemic parameters, the duration of diabetes and HbA1c level were significant predictors of visual outcomes in our study cohort. In addition, our analysis of ophthalmological parameters revealed that macular tractional detachment had a significant negative effect on the long-term preservation of visual acuity. Therefore, early vitrectomy can be considered a useful tool to provide patients with the necessary time to achieve good metabolic control and maintain a high quality of life and working ability during their active years.

## Figures and Tables

**Table 1:** Baseline characteristics of our patients. PPV: pars plana vitrectomy; CV: cardiovascular; DM: diabetes mellitus, MRD: macular tractional detachment, TRD: tractional retinal detachment elsewhere, ns: not significant (Mann–Whitney U test was applied for comparison of type 1 and type 2 patients’ non-categorical parameters, while Fisher’s exact test was used for analysis of categorical data).

	Overall	Type 1	Type 2	*p*-Value
General characteristics				
Number (female/male)	66 (22/45)	40 (15/25)	26 (19/7)	ns
Age, years	52 (21–80)	45 (21–78)	58 (45–80)	**<0.001**
Follow-up after PPV on first eye, months (mean min–max)	38 (9–125)	32.5 (9–125)	38.5 (9–102)	ns
Duration of DM, years	20 (0–57)	24 (0–50)	15 (2–57)	**<0.001**
Systemic characteristics				
HbA1c (%) at time of first PPV (mean, min–max)	7.3 (5.5–13.5)	7.8 (5.7–12.4)	7 (5.5–11.6)	**0.029**
HbA1c (%) at end of follow-up (mean, min–max)	7.3 (5.1–17.0)	7.6 (5.1–17)	6.9 (5.4–9.2)	**0.004**
Mean HbA1c (%) (mean, min–max)	7.5 (5.4–13.1)	7.7 (5.4–13.1)	7.2 (5.6–10.4)	**0.002**
Renal insufficiency (patients)	9	7	2	ns
Severe CV complications (patients)	17	10	7	ns
Unstable blood pressure (patients)	18	12	6	ns
Visual acuity				
BCVA at first attendance (mean min–max)	0.05 (0.001–1)	0.1 (0.005–1.0)	0.04 (0.01–1.0)	ns
Good BCVA at first attendance (eyes)	38	27	11	ns
Legally blind at first attendance (eyes)	86	46	40	ns
Preoperative ocular factors				
Iris rubeosis at first attendance (eyes)	9	7	2	ns
Glaucoma at first attendance (eyes)	11	5	6	ns
MRD before PPV (eyes)	44	29	15	ns
TRD before PPV (eyes)	46	28	18	ns
Vitreous hemorrhage (eyes)	85	49	36	ns
Preoperative anti-VEGF injections (eyes)	41	29	12	ns

**Table 2:** Changes in anatomical findings in the three study groups. (Favorable functional outcome: 0.5 ≤ BCVA (eyes), legally blind: BCVA ≤ 0.2.)

Parameters at First/at Final (No. of Eyes)	All Eyes (n = 132)	First Eyes (n = 66)	Fellow Eyes with PPV (n = 31)	Fellow Eyes without PPV (n = 35)
BCVA Good at first/favorable at final	38/53	1/21	13/10	24/24
Legally blind eyes	80/61	58/38	13/17	13/10
Tractional retinal detachment	47/9	27/4	18/3	3/3
Macular tractional detachment	44/2	28/9	14/0	2/2
Posterior hyaloid detachment	29/-	24/-	5/-	-
Silicone oil usage	-/58	-/38	-/20	-
Intraoperative complication	-/2	-/2	-/0	-
Postop. vitreous hemorrhage	-/21	-/17	-/4	-
Neovascularization on iris	9/10	5/6	4/4	-
Glaucoma	11/39	7/26	4/13	-
Refractory stage glaucoma	0/10	0/5	0/5	-

**Table 3:** Results of Spearman rank correlation (ns, non-significant). Parameters listed below might have impact on development of BCVA.

Favorable BCVA at Final		
**Parameter**	**Correlation Coefficient**	***p* Value**
Age	0.246	0.004
Type of DM	−0.028	ns
Duration of DM	0.181	0.037
HbA1c at baseline	−0.144	ns
HbA1c at final	−0.162	ns
HbA1c, average	−0.173	0.047
Renal failure	0.035	ns
Severe CV complication	−0.129	ns
Unstable blood pressure	−0.189	0.030
Follow-up	0.039	ns
Fellow eye in severe condition	0.201	0.021
Legal blindness at start	−0.406	0.000
Good BCVA at start	0.469	0.000
First BCVA	0.404	0.000
Rubeosis	−0.222	0.011
Preoperative secondary glaucoma	−0.247	0.004
Vitreous hemorrhage	0.023	ns
Tractional macular detachment	−0.448	0.000
Posterior hyaloid detachment	0.355	0.018
Vitrectomy must be performed	−0.313	<0.001
Phaco-combined vitrectomy	−0.236	0.006
Silicon oil was used	−0.417	0.000
Number of PPV	−0.279	0.001
Preoperative use of anti-VEGF	0.120	ns

## Data Availability

The datasets are available: doi:10.5061/dryad.4b8gthtnj.

## Supplementary Materials

The following supporting information can be downloaded at https://www.mdpi.com/article/10.3390/life14080993/s1, Table S1: The literature data on the outcome of pars plana vitrectomy for proliferative diabetic retinopathy. References [7,10,12,15,21,24,25,26,27,28,29,30,31,32,33,34,35] are cited in Supplementary Materials.
