# Supplementary material for: Personalized Management of Patients with Proliferative Diabetic Vitreoretinopathy
Source: Life (Basel). 2024 Aug 9;14(8):993. doi: 10.3390/life14080993 (PMC11355517; doi:10.3390/life14080993)
Supplement: Supplementary file 1 [file life-14-00993-s001.zip › life-3131089-supplementary.pdf]

Table S1. Literature data on the outcome of pars plana vitrectomy for proliferative diabetic retinopathy.

Study/Author, year, study type	Reference	Plan, details	Outcome
PDR- randomized prospective clinical trials			
**drcr.net Protocol N**	[24]	PPVs after ranibizumab vs. saline<br>Number of eyes/patients: 261/261<br>Follow up (months): 12	In cases of PDR related vitreous hemorrhages, no difference was showed on the rate of PPVs by 16 weeks after intravitreal ranibizumab and saline administration. However, the advantages of short term effect of ranibizumab were confirmed (imporved VA, less recurrent VHs, higher proportion of completed PRP).
**drcr.net Protocol S**	[25, 26]	PRP vs. Ranibizumab<br>Number of eyes /number of patients: 394 /305<br>Follow up (months): 60	At 2 years: ranibizumab proved to be non-inferior to PRP for change in VA from baseline, and also superior gain could be achieved in VA.<br>At 5 years, there was no difference in mean VA change, but more complications were described in PRP group (peripheral visual defect, DME, retinal detachment).
**DRVS**	[10]	Early vs. deferred PPV<br>Number of eyes / number of patients: 616 /616<br>Follow up (months): 48	In patients with type 1 diabetes, better VA can be achieved with early vitrectomy.
**drcr.net Protocol AB**	[12]	Aflibercept vs prompt PPV with PRP for VH<br>Number of eyes /number of patients: 205 /205)<br>Follow up (months): 24	No significant difference in VA outcomes at 2 years. Higher rate of recurrent VH and persistent NV in aflibercept group.
**CLARITY**	[27]	PRP vs. Aflibercept<br>Number of eyes /number of patients: 616 /232)<br>Follow up (months): 12	Aflibercept was found superior to PRP in VA improvement at 1 year.<br>DME, VH, need for vitrectomy, visual loss were more frequent in PRP group.<br>Earlier and more complete NV regression was achieved with aflibercept.
PDR- retro/prospective studies			
**Khan et al,**<br>2021<br>retrospective	[28]	Long term outcomes og PPV<br>Number of eyes /number of patients:1038 /519)<br>Follow up (months): 131	After complete PRP, 1/3 of the study cohort needed vitrectomies.<br>Independent predictors: previous cataract surgery, poor baseline visual acuity.
**Berrocal et al,**<br>2022<br>retrospective	[7]	Conventional treatment vs. PPV<br>Number of eyes /number of patients:128 /64<br>Follow up (months): min. 96	Eyes that underwent PPV had better VA outcomes than eyes receiving conventional treatment.
**Motoda et al,**<br>2018	[21]	Perioperative variables	Duration of operation was the only significant variable associated with postoperative VH.

retrospective		Number of eyes /number of patients: 72 /64<br>Follow up (months): 12	(other examined factors: treatment with antiplatelet and antihypertesive drugs, preoperative HbA1c, BMI, CV disease, concomitant cataract surgery)
**Patel et al,**<br>2023<br>retrospective	[29]	Perioperative variables<br><br>Number of eyes /number of patients: 81 /81<br>Follow up (months): 17.7 (median)	Preoperative PRP and anti-VEGF injections resulted in lower rates of postoperative VH.<br><br>Longer duration of diabetes and PDR were inversely correlated with BCVA at 12 months.<br>Higher preoperative HbA1c levels were associated with higher incidence of postoperative VHs.
**Schreur et al,**<br>2021<br>retrospective	[30]	Long term outcomes of PPV<br><br>Number of eyes /number of patients: 217x2 /217<br>Follow up (months): 6-120	Majority of patients retained functional VA (>0.3) in at least 1 eye.<br><br>Vision loss in the fellow eye is a predictive factor for poor prognosis (VA and re-PPV).<br><br>Vitrectomy of the fellow eye associated with shorter DM duration, worse contralateral VA, higher HbA1c level, worse DR severity of the fellow eye.
**Gupta et al,**<br>2012<br>retrospective	[31]	Visual outcomes of PPV, predictive factors<br><br>Number of eyes /number of patients: 185 /158<br>Follow up (months): 12	BCVA improved by at least 3 ETDRS lines.<br><br>Poor predictors of VA success: diabetes duration, use of insulin, ischaemic heart disease, delay in surgery, missed appointments.
**Liao et al,**<br>2020<br>retrospective	[32]	Outcomes of PPV in young patients vs seniors<br><br>Number of eyes /number of patients: 116 /92<br>Follow up (months): min. 24 months	Younger patients (18-44 years old): limited and worse outcomes, and also higher incidence of neovascular glaucoma.
**Sato et al,**<br>2017<br>retrospective	[33]	Predictors of PVH<br><br>Number of eyes /number of patients: 106 /78<br>Follow up (months): 6-31 months (mean: 11.9 months)	Incidences of early and late PVH: 18.9% and 17.9%.<br>Most important factor for the development of early PVH was intraoperative bleeding from NVD, and for the developmenet of late PVH: higher HbA1c.

**Yorston**<br>2008<br>prospective	[34]	Predictors of visual outcome after PPV<br><br>Number of eyes /number of patients: 174 /148<br>Follow up (months): min. 4 months	>70% of eyes will regain vision of 0.1 or better.<br><br>Independent risk factors for poor postoperative VA: preoperative VA, macular detachment, long-acting intraocular tamponade.
**Tandias et al,**<br>2022<br>retrospective	[35]	Stage of PVD as prognostic factor<br><br>Number of eyes /number of patients: 136 /117<br>Follow up (months): 12	No PVD: higher incidence of postoperative hypotony, TRD, higher rate of re-PPV, poorer BCVA.<br>Complete PVD at baseline: improved postoperative VA at 6 months.
**McCullough et al,**<br>2023<br>meta-analysis	[15]	PPV in PDR eyes with TRD<br><br>Number of eyes: 3839<br>(38 studies)	Higher preoperative VA was the only factor associated with higher postoperative vision,<br>early intervention should be considered.<br><br>However in PDR patients with TRD overall, final postoperative VA remains low.
